# Transcriptome Analysis Reveals the Important Role of Vitamin B_12_ in the Response of *Natronorubrum daqingense* to Salt Stress

**DOI:** 10.3390/ijms25084168

**Published:** 2024-04-10

**Authors:** Qi Wang, Zhiwei Wang, Jiaqi Guan, Jinzhu Song

**Affiliations:** School of Life Science and Technology, Harbin Institute of Technology, Harbin 150080, China; 19b928027@stu.hit.edu.cn (Q.W.); wangzhiwei0821@163.com (Z.W.); g13610013008@163.com (J.G.)

**Keywords:** *Natronorubrum daqingense*, salt stress, vitamin B_12_, extremely halophilic archaea, transcriptome analysis

## Abstract

*Natronorubrum daqingense* JX313^T^ is an extremely halophilic archaea that can grow in a NaCl-saturated environment. The excellent salt tolerance of *N. daqingense* makes it a high-potential candidate for researching the salt stress mechanisms of halophilic microorganisms from *Natronorubrum*. In this study, transcriptome analysis revealed that three genes related to the biosynthesis of vitamin B_12_ were upregulated in response to salt stress. For the wild-type (WT) strain JX313^T^, the low-salt adaptive mutant LND5, and the vitamin B_12_ synthesis-deficient strain Δ*cobC*, the exogenous addition of 10 mg/L of vitamin B_12_ could maximize their cell survival and biomass in both optimal and salt stress environments. Knockout of *cobC* resulted in changes in the growth boundary of the strain, as well as a significant decrease in cell survival and biomass, and the inability to synthesize vitamin B_12_. According to the HPLC analysis, when the external NaCl concentration (*w*/*v*) increased from 17.5% (optimal) to 22.5% (5% salt stress), the intracellular accumulation of vitamin B_12_ in WT increased significantly from (11.54 ± 0.44) mg/L to (15.23 ± 0.20) mg/L. In summary, *N. daqingense* is capable of absorbing or synthesizing vitamin B_12_ in response to salt stress, suggesting that vitamin B_12_ serves as a specific compatible solute effector for *N. daqingense* during salt stress.

## 1. Introduction

Salt stress, characterized by high-salinity conditions, poses significant challenges to microbial physiology, affecting cellular processes such as protein function and membrane integrity [1,2]. Microorganisms have evolved various tolerance mechanisms: (1) Halophilic microorganisms use Na^+^/H^+^ exchangers (NHEs) [3] to achieve Na^+^ efflux and accumulate H^+^ or K^+^ intracellularly to maintain osmotic balance. The secondary Na^+^/H^+^ pumps, named Na^+^/H^+^ antiporters, are the principal Na^+^ efflux system of halophilic microorganisms and serve as one of the main adaptive response mechanisms to Na^+^ stress [4]. (2) Microorganisms modify the cellular membrane permeability by adjusting the composition and ratio of membrane lipid constituents within cell membranes to enhance their adaptability to high-salinity environments [5,6]. (3) Accumulation of compatible solutes is one of the main mechanisms by which halophilic microorganisms tolerate osmotic stress, which is mainly achieved by ingesting compatible solutes from a medium or synthesizing them intracellularly. These compatible solutes can maintain the positive turgor pressure environment required for cell division [7].

Most microorganisms have not evolved extensive genetic changes to adapt to high-salinity environments. As their cytoplasm is unable to tolerate salt, the accumulation of organic or inorganic compatible solutes has become a widely used strategy to adapt to osmotic changes, and most microorganisms rely exclusively on this strategy for osmotic adaption [8]. Various organic compounds from different classes have been proven to act as osmotic regulators in different microbial communities, including betaine, polyols, ectoine, sugars, amino acids, N-derivatized carboxamides of glutamine, and N-acetylated diamino acids [9,10,11,12,13]. Within numerous extremophiles, the accumulation of these small-molecule compounds is not limited to salt stress; moreover, this effect occurs as a reaction to other environmental stressors, including temperature stress [14,15,16,17].

Vitamin B_12_, a familiar hydrosoluble vitamin, also named cobalamin, is one of the most complex small-molecule natural compounds. De novo biosynthesis of vitamin B_12_ is exclusive to certain bacteria and fungi, which usually exist in soil, water, animals, and plants [18]. Therefore, microorganisms play an important role as synthesizers and transmitters of vitamin B_12_ in nature [19]. The biosynthesis of vitamin B_12_ is a complex process involving about 30 enzymatic steps, and the genes involved are often prefixed with *cob* or *cbi*, depending on whether the pathway is oxygen-dependent or oxygen-independent [20]. Vitamin B_12_ is important to microbial metabolism, taking part in various reactions such as acetyl-CoA synthesis, methyl transfer in methane-producing archaea, ribonucleotide reductase and vitamin B_12_-dependent fermentation processes in enteric bacteria [21,22,23,24]. Vitamin B_12_ is essential for human health, involved in the metabolism of proteins, fats and carbohydrates [25,26]. It also supports the proper functioning of the nervous system, helps regulate insulin secretion and enhances insulin sensitivity [27,28]. Vitamin B_12_ is mainly found in meat, fish, eggs and dairy products, and can also be consumed through supplements [29,30]. Vitamin B_12_ also plays an important role in the resistance of microbes and plant cells to stress; for example, the biosynthesis of vitamin B_12_ is necessary for *Listeria monocytogenes* growth under low-temperature stress and copper stress [31]. Bo Xie et al. [32] revealed that vitamin B_12_-producing bacteria can increase the fitness of the unicellular alga *Chlamydomonas reinhardtii* following an increase in environmental temperature. Hamed Kesharvarz et al. [33] have shown that priming vitamin B_12_ into seeds can enhance the resistance to salinity of *Phaseolus vulgaris* L. Moreover, exogenously added vitamin B_12_ could promote maize seed germination and growth under low-temperature stress [34].

*Natronorubrum daqingense* JX313^T^ is an extremely halophilic archaeon isolated from highly salinized soil. It is Gram-negative and capable of growth in environments with NaCl concentrations (*w*/*v*) ranging from 10% to salt saturation (optimal at 17.5%) and pH ranging from 8.0 to 11.0 (optimal at 10.0) [35]. *N. daqingense* was initially classified under the genus *Haloterrigena*, and its whole genome sequencing data were published in 2021 [36]. Later, de la Haba et al. [37] conducted a reanalysis of the phylogenetic evolution of *Haloterrigena*, *Natrinema* and other neighboring genera. According to the results, they suggested reclassifying and renaming *Haloterrigena daqingensis* to *N. daqingense*. In our previous study, we characterized two novel NhaC-type Na^+^(K^+^, Li^+^)/H^+^ antiporters from *N. daqingense*, providing a foundation for researching its Na^+^ efflux strategies related to salt stress [38]. In this study, based on transcriptome sequencing, differentially expressed genes of *N. daqingense* JX313^T^ were analyzed in an optimal environment and under an additional 5% salt stress environment. Their results revealed the important role of vitamin B_12_ in the response of *N. daqingense* to salt stress. The constructed low-salt adaptive mutant and vitamin B_12_ synthesis-deficient strains, combined with the detected intracellular vitamin B_12_ content and the changes in cell survival and biomass after exogenous vitamin B_12_ addition, suggest that vitamin B_12_ serves as an alternative compatible solute effector in the response of *N. daqingense* to salt stress, which can be absorbed from the environment or synthesized and accumulated intracellularly to resist osmotic stress.

## 2. Results

### 2.1. Transcriptome Sequencing and Analysis of Differentially Expressed Genes

**Figure 1:**
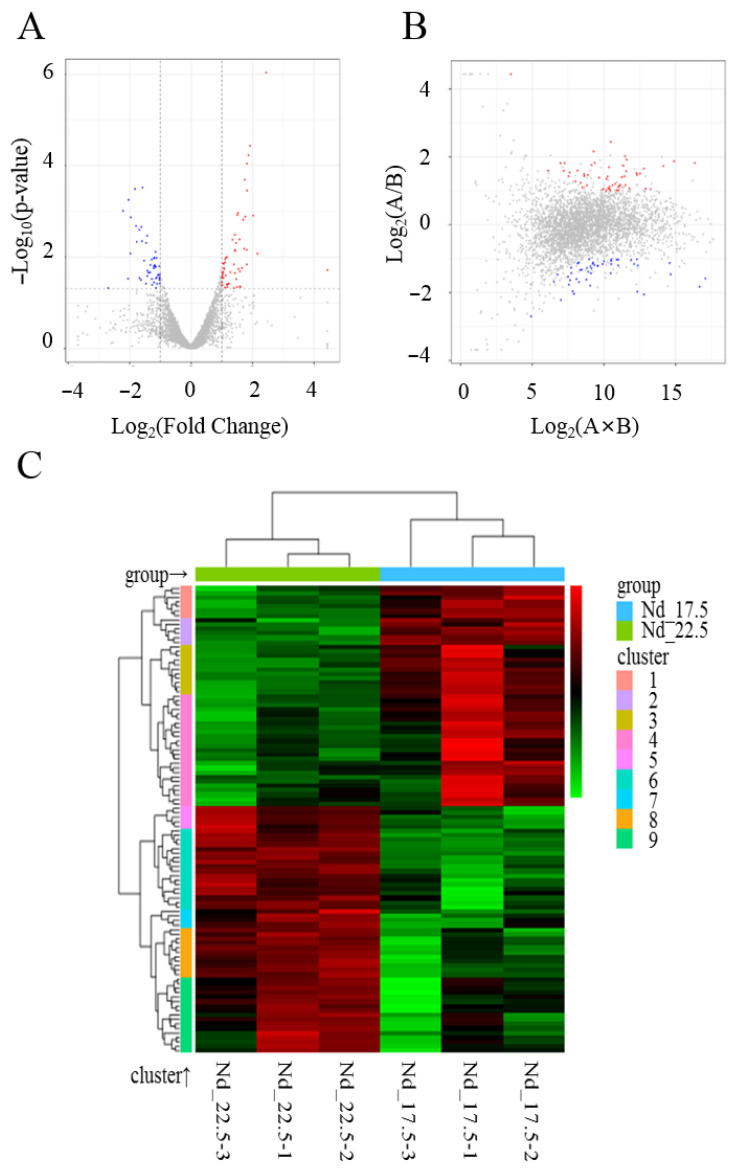
Analysis of *N. daiqngense* differentially expressed genes (DEGs) under salt stress; the control group is the optimal environment, while the treatment group is the additional 5% salt stress environment. (**A**) Volcano plot of DEGs under salt stress; blue, red, and gray dots, respectively, represent downregulated genes, upregulated genes, and genes with non-significant differential expression. (**B**) M versus A plot of DEGs under salt stress; A and B, respectively represent the gene expression levels in the two samples, and the color correspondence of the dots is the same as described above. (**C**) Clustering of DEGs; red represents upregulated genes and green represents downregulated genes.

Transcriptome sequencing was performed to analyze the differentially expressed genes (DEGs), with *N. daqingense* in the optimal environment as the control group (Nd_17.5) and *N. daqingense* in the additional 5% salt stress environment as the treatment group (Nd_22.5). A total of 104 DEGs were obtained, including 55 upregulated genes and 49 downregulated genes. The calculation of fold change was based on Nd_22.5/Nd_17.5, with the conditions for selecting DEGs being |log_2_ (Fold Change)| > 1 and *p* < 0.05. DEGs were plotted in a volcano plot and M versus A (MA) plot, as shown in Figure 1A,B, with gene information (partial) shown in Table 1. The Euclidean method was used to calculate the distance, and complete linkage was used to conduct the biclustering analysis. The 104 DEGs were clustered into nine clusters, as shown in Figure 1C.

**Figure 2:**
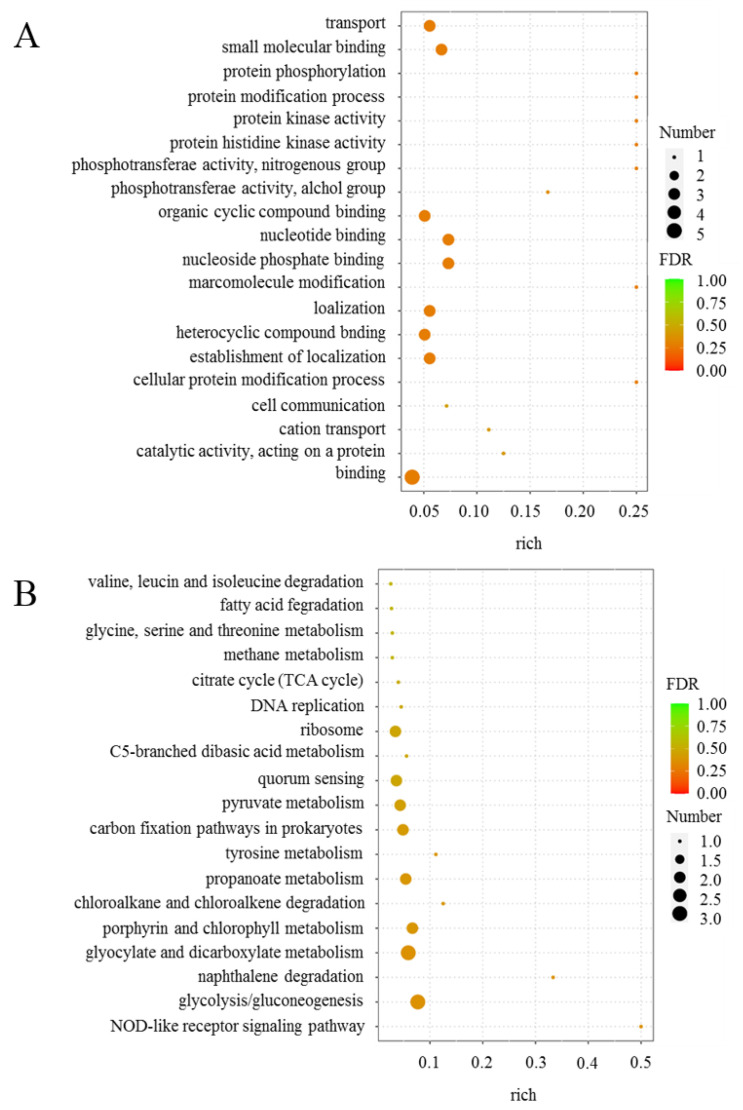
Functional enrichment analysis of *N. daiqngense* DEGs (FDR stands for false discovery rate). (**A**) GO enrichment analysis bubble plot; (**B**) KEGG enrichment analysis bubble plot.

The top 20 gene ontology (GO) terms with the smallest false discovery rates (FDRs) are shown in Figure 2A. Among the GO enrichment results of DEGs caused by salt stress, the highly enriched and more significant terms were binding, cellular protein modification process, the establishment of localization, heterocycle compound binding, localization, nucleoside phosphate binding, nucleoside binding, organic cyclic compound binding, small molecular binding, and transport. As shown in Figure 2B, among the 19 Kyoto Encyclopedia of Genes and Genomes (KEGG) pathways enriched by the DEGs caused by salt stress, the highly enriched and significant terms were glycolysis/gluconeogenesis, glyocylate and dicarboxylate metabolism, porphyrin and chloroalkene degradation, propanoate metabolism, pyruvate metabolism, carbon fixation pathways in prokaryotes, quorum sensing, the ribosome pathway, etc. In this study, we focused on the changes in genes related to vitamin B_12_ synthesis (as shown in Table 1), which may suggest the important role of vitamin B_12_ in *N. daqingense* resistance to salt stress.

### 2.2. Ultraviolet Mutagenesis of Low-Salt Adaptive Mutant LND5

**Figure 3:**
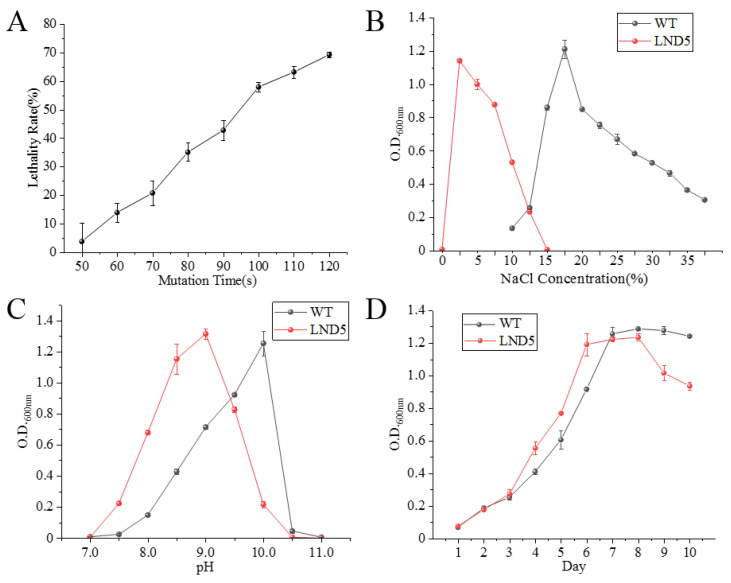
Ultraviolet mutagenesis of *N. daiqngense* low-salt adaptive mutant LND5 and detection of physiological characteristics. (**A**) Ultraviolet mutagenesis lethality curve; (**B**) detection of the optimal NaCl concentration of LND5; (**C**) detection of the optimal pH of LND5; and (**D**) growth curves of WT and LND5 in their respective optimal environments.

As shown in Figure 3A, when the UV irradiation time was 120 s, the lethality rate of the strain was 69.72%. Generally, the mutagenesis effect is optimal when the lethality rate is around 70%, then the subsequent mutagenesis time is set to 120 s. The effect of NaCl concentration (*w*/*v*) on the obtained optimal low-salt adaptive mutant LND5 is shown in Figure 3B. LND5 could not grow normally in the medium lacking NaCl; when the NaCl concentration exceeded 10%, the growth of LND5 was significantly inhibited and almost completely halted at 15%. As shown in Figure 3B, compared with the wild-type (WT) strain JX313^T^, LND5 could grow when the NaCl concentration was lower than 10% and exhibited optimal growth when the NaCl concentration was 2.5%, which was five times lower than that of the WT strain. The tolerance of LND5 to the pH of the culture environment ranged from 7.5 to 10.5, the tolerance of LND5 to the strong alkaline conditions was reduced compared with WT, and the optimal pH decreased from 10.0 to 9.0, as shown in Figure 3C. The growth curve of LND5 was measured in the medium containing 2.5% NaCl and pH 9.0, as shown in Figure 3D. LND5 entered the logarithmic phase on the third day and reached the stationary phase on the sixth day. The biomass of LND5 peaked on the eighth day and then entered the decline phase. Therefore, related indicators should be measured on the seventh day in subsequent experiments. Compared with the WT in the optimal environment, the biomass of LND5 during the logarithmic phase was higher in its optimal growth environment. When LND5 entered the decline phase, its biomass decreased more rapidly than that of the WT. No significant difference was found in the maximum biomass that can be achieved using both WT and LND5 during the growth process.

### 2.3. Construction of ΔcobC and +cobC

**Figure 4:**
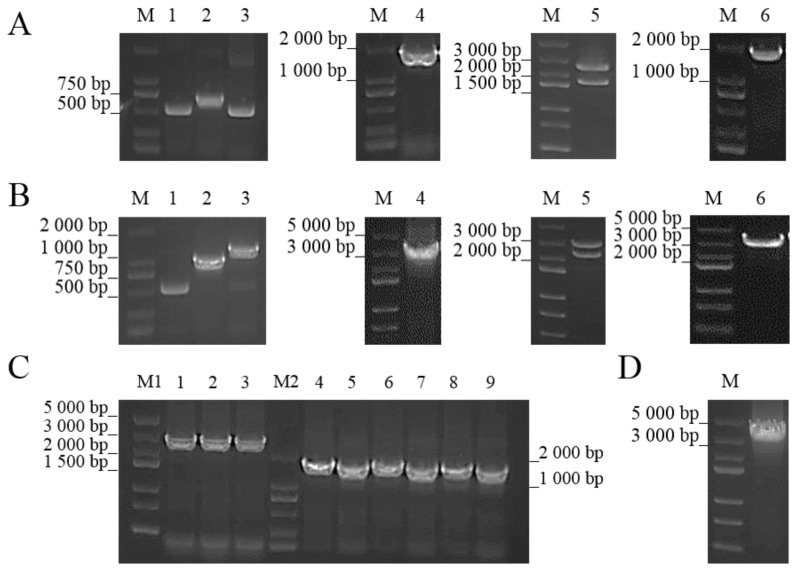
Construction of *N. daiqngense* Δ*cobC* and +*cobC*. (**A**) Preparation of the linear DNA fragment for *cobC* knockout. Lanes 1–6 contain the following in order: *cobC* upstream homology arm, erythromycin resistance fragment, *cobC* downstream homology arm, PCR product of pUC-*cobC*-ko via M13-47F/M13R primers, double digestion product of pUC-*cobC*-ko with *Eco*RI and *Hind*III, and purified linear DNA fragment for *cobC* knockout. (**B**) Preparation of the linear DNA fragment for *cobC* complementation. Lanes 1–6 contain the following in order: *cobC* upstream homology arm, hygromycin resistance fragment, *cobC* and downstream homology arm, PCR product of pUC-*cobC*-c via M13-47F/M13R primers, digestion product of pUC-*cobC*-c with *Bam*HI, and purified linear DNA fragment for *cobC* complementation. (**C**) Verification of *cobC* knockout. Lanes 1–3 contain PCR products of gDNA via VY-FP/VY-RP primers; lanes 4, 6, and 8 contain PCR products of gDNA via VY-FP/VE-RP primers; and lanes 5, 7, and 9 contain PCR products of gDNA via VE-FP/VY-RP. (**D**) Verification of *cobC* complementation; the lane contains the PCR product of gDNA via VY-FP/VY-RP primers.

As shown in Figure 4A,B, recombinant plasmids pUC-*cobC*-ko and pUC-*cobC*-c, containing linear DNA fragments for *cobC* knockout and complementation, were constructed by using homologous recombination, and corresponding high-purity linear fragments were prepared. To validate the knockout or complementation of *cobC*, primers VY-FP/VY-RP were designed for sequencing validation at 750 bp upstream and 740 bp downstream of the gene, respectively. The length of *cobC* is 717 bp, which was close to the erythromycin resistance sequence (735 bp). Therefore, the gDNA from the strain (Erm^+^) obtained after protoplast transformation was PCR amplified with three pairs of primers, VY-FP/VY-RP, VY-FP/VE-RP, and VE-FP/VY-RP, as shown in Figure 4C, respectively, and sequencing data confirmed the knockout of *cobC*. As shown in Figure 4D, the gDNA from the strain (Hyg^+^) obtained after protoplast transformation was PCR amplified with primers VY-FP/VY-RP, and combined with sequencing data, *cobC* was complimented.

**Figure 5:**
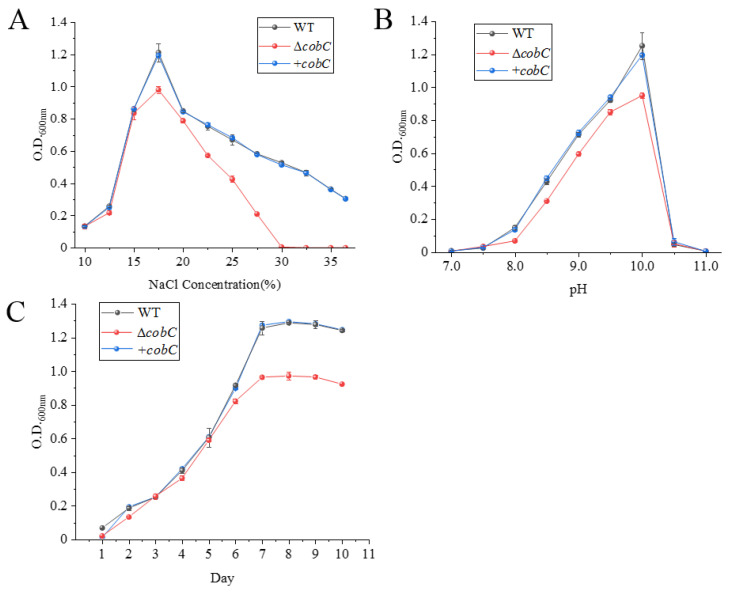
Detection of the physiological characteristics of *N. daiqngense* Δ*cobC* and +*cobC*. (**A**) Detection of the optimal NaCl concentration of Δ*cobC* and +*cobC*; (**B**) detection of the optimal pH of Δ*cobC* and +*cobC*; and (**C**) growth curve of WT, Δ*cobC,* and +*cobC*.

As shown in Figure 5A, both Δ*cobC* and WT were unable to grow when the NaCl concentration (*w*/*v*) was below 10%, and both reached the maximum biomass when the NaCl concentration was 17.5%. However, the biomass of Δ*cobC* reduced significantly compared with WT. Δ*cobC* was unable to grow when the NaCl concentration exceeded 30%, whereas the WT could still grow when the NaCl saturation was reached. As shown in Figure 5B, both Δ*cobC* and WT could tolerate a pH range of 8.0–11.0, and both reached the maximum biomass when the pH was 10.0. Δ*cobC* entered the logarithmic phase on the second day and reached the stationary phase on the seventh day, as shown in Figure 5C. Therefore, the seventh day should be chosen for the measurement of related indicators in the subsequent experiments. The biomass of Δ*cobC* was mostly lower than that of WT during the logarithmic phase, and the biomass significantly decreased compared with WT after entering the stationary phase. Furthermore, the growth curves of the WT and +*cobC* were essentially consistent, with no significant differences. Combining the data from Figure 5A,B, the effects of NaCl concentration and pH on the biomass of +*cobC* were generally consistent with those of WT, indicating that the phenotypic changes in Δ*cobC* are caused by the knockout of *cobC*, rather than by non-specific factors.

### 2.4. Effects of Exogenous Vitamin B_12_ Addition

**Figure 6:**
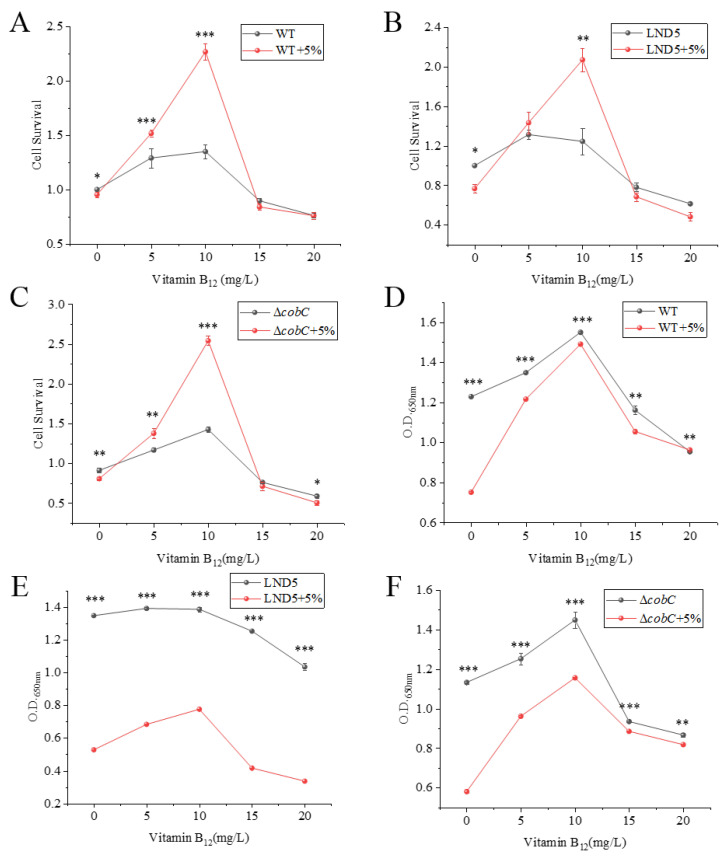
The effect of exogenous addition of vitamin B_12_. (**A**–**C**) The effect of exogenous addition of vitamin B_12_ on the cell survival of *N. daiqngense* JX313^T^ (WT), LND5, and Δ*cobC*. (**D**–**F**) The effect of the exogenous addition of vitamin B_12_ on the biomass of *N. daiqngense* JX313^T^ (WT), LND5, and Δ*cobC*; the significance analysis represents the comparison between the optimal environment and the salt stress environment. *** represents *p* < 0.001, ** represents *p* < 0.01, * represents *p* < 0.05, and no mark represents non-significant.

As shown in Figure 6A–C, the cell survival of WT, LND5, and Δ*cobC* under 5% salt stress was significantly reduced compared with optimal conditions. Overall, the exogenous addition of vitamin B_12_ at a lower concentration resulted in a pronounced positive effect on the cell survival of strains under salt stress, and even the cell survival was higher than that of the strains in the optimal environments at the same concentration. The most significant enhancement was observed when the addition of vitamin B_12_ reached 10 mg/L. When the concentration of exogenously added vitamin B_12_ exceeded 10 mg/L, the cell survival of strains was inhibited. As shown in Figure 6D–F, when the concentration of exogenously added vitamin B_12_ was low, the biomass of strains except LND5 in the optimal environments was significantly promoted, while the enhancement effect was significant for strains under salt stress. When the exogenous addition of vitamin B_12_ was 10 mg/L, the biomass of strains reached the maximum, and the biomass of WT under salt stress recovered to the optimal environment closely. When the concentration of exogenously added vitamin B_12_ exceeded 10 mg/L, the biomass of the strains was inhibited. As shown in Figure 6, the exogenous addition of appropriate concentrations of vitamin B_12_ helps to enhance the cell survival and biomass of *N. daqingense* under salt stress, indicating that *N. daqingense* can tolerate salt stress by taking up vitamin B_12_ from the culture environment. Taken together, it is speculated that *N. daqingense* can absorb vitamin B_12_ from the environment and accumulate it intracellularly to resist salt stress, and vitamin B_12_ is likely to be a specific compatible solute effector in this process.

### 2.5. Detection of Intracellular Vitamin B_12_ Content

**Figure 7:**
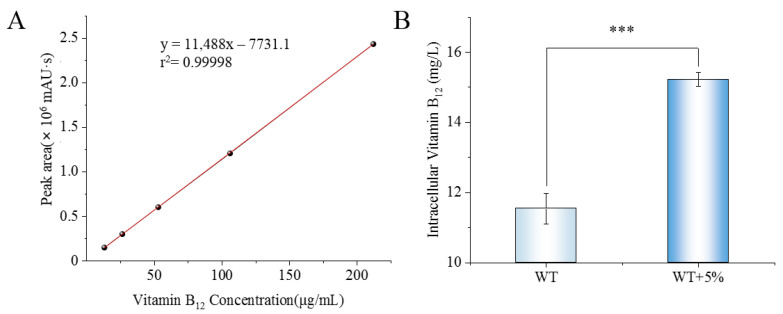
Detection of intracellular vitamin B_12_ content. (**A**) Vitamin B_12_ standard curve. (**B**) The effect of salt stress on intracellular vitamin B_12_ content; the significance analysis represents the comparison between the optimal environment and the salt stress environment. *** indicates that the analysis of significant difference between the two groups is *p* < 0.001.

To investigate whether *N. daqingense* can synthesize and accumulate vitamin B_12_ intracellularly to resist salt stress, this study measured the intracellular vitamin B_12_ content of WT and Δ*cobC* in both optimal and salt stress environments using HPLC. The standard curve correlating the concentration of vitamin B_12_ standards with the peak area is shown in Figure 7A, and the intracellular content of vitamin B_12_ per liter of fermentation broth was calculated according to the fitting equation and the dilution factor from the “bacterial milking” described in Section 4.6. According to the HPLC results, vitamin B_12_ was not detected in Δ*cobC* from either the optimal or salt stress environments, which indicated the knockout of *cobC* made *N. daqingense* unable to synthesize vitamin B_12_, and the results for WT are shown in Figure 7B. In the optimal environment, the intracellular vitamin B_12_ content of WT of fermentation broth was (11.54 ± 0.44) mg/L; when an additional 5% salt stress was applied in the medium, the intracellular vitamin B_12_ significantly increased to (15.23 ± 0.20) mg/L. Taken together, *N. daqingense* can synthesize and accumulate vitamin B_12_ intracellularly to resist salt stress, and vitamin B_12_ is likely to be a specific compatible solute effector in this process.

## 3. Discussion

Compatible solutes can act as osmotic protectants, alleviating the inhibitory effects of high osmotic stress on microorganisms when added to a medium. These compatible solutes can provide osmotic protection via uptake from the medium or via de novo synthesis and intracellular accumulation [39]. Glucosylglycerol is present at the reducing terminus of the polysaccharides in *Bifidobacterium* and exists in free form in a few mesophilic bacteria and thermophilic archaea. In recent years, glucosylglycerol has been identified in some microorganisms as an accumulable compatible solute to tolerate salt stress or nitrogen nutrient deficiency [40]. In a study of compatible solutes of *Spiribacter salinus*, León et al. [41] found that the intracellular content of ectoine increased from a basal level of 80 μM to 170 μM under conditions ranging from 0.6 M NaCl to the optimal concentration of 0.8 M NaCl. Although further increasing the NaCl concentration to 1.3 M significantly reduced the biomass, there was no corresponding increase in the intracellular content of ectoine, which only increased when the NaCl concentration exceeded 1.6 M. Betaine can act as a compatible solute in most microorganisms, and its concentration varies with the external NaCl concentration. Studies have shown that when the external NaCl concentrations were 0.51 M, 1.7 M, and 3.4 M, the intracellular concentrations of betaine were (0.21 ± 0.2) M, (0.65 ± 0.06) M, and (0.97 ± 0.09) M [42]. Moreover, trehalose was shown to act as a compatible solute for *Chromohalobacter israelensis* when the external NaCl concentration was below 0.6 M [43]. Trehalose is also one of the major compatible solutes of *Desulfovibrio halophilus*; when the medium contains 2.5 M NaCl without a betaine source, the strain can accumulate 8 μM/mg of protein and about 2.5 μM/mg of protein intracellularly [44].

In this study, transcriptome sequencing analysis revealed that three genes related to vitamin B_12_ biosynthesis were upregulated by salt stress, as shown in Table 1. The gene *cbiE*, which encodes for cobalt–precorrin-7-[C(5)]-methyltransferase, catalyzes the conversion of cobalt–precorrin 7 to cobalt–precorrin 8. The gene *cobH*, which encodes for precorrin 8X methylmutase, catalyzes the conversion of precorrin 8X to hydrogenobyrinate. The gene *cobC*, which encodes for cobalamin biosynthesis protein, catalyzes the conversion of adenosyl–GDP–cobinamide to cobalamin [45,46]. In current research on halophilic microorganisms, vitamin B_12_ typically acts as a growth factor. Some vitamin B_12_-dependent microorganisms may have growth restriction or complete cessation in the absence of vitamin B_12_. However, the exogenous addition of vitamin B_12_ (50 μg/L) to *Methylophaga lonarensis*, a methanogenic halophile isolated from a saline lake, can promote its growth at higher salinity, although this strain is not a vitamin B_12_-dependent strain [47]. In this study, preliminary experiments showed that adding trace amounts of vitamin B_12_ (measured in μg/L) to the medium had no significant effect on biomass. As shown in Figure 6, when the exogenous addition of vitamin B_12_ was at 10 mg/L, WT, LND5, and Δ*cobC* all achieved the highest cell survival and biomass under the optimal and salt stress environments. Moreover, the intracellular vitamin B_12_ concentration of WT increased, and the Δ*cobC* growth boundary, cell survival, and biomass changed under salt stress. These results indicated that *N. daqingense* did not use vitamin B_12_ as a regulatory factor to participate in other metabolic pathways, but instead ingested vitamin B_12_ from the medium as a compatible solute or an effector to maintain osmotic balance [8].

In this study, we detected that the intracellular vitamin B_12_ accumulated by *N. daqingense* under salt stress can reach (15.23 ± 0.20) mg/L, which shows a certain development potential compared with other vitamin B_12_-producing strains [19]. Future studies will focus on the role of vitamin B_12_ in the response of closely related species to salt stress and analyze the molecular mechanism of vitamin B_12_ acting as a compatible solute effector in the response of *N. daqingense* to salt stress. Studies of the relationship between the biosynthesis of vitamin B_12_ and other salt-stress-related genes in *N. daqingense*, as well as the transcriptome sequencing of the low-salt adaptive mutant LND5, can further elucidate the mechanisms of *N. daqingense* in response to salt stress. Combined with the genetic modification and optimization of fermentation conditions, *N. daiqngense* can be utilized for the high production of vitamin B_12_.

## 4. Materials and Methods

### 4.1. Strains, Plasmids and Growth Conditions

The strains and plasmids used in the current study are detailed in Table 2. High-salt Luria Bertani (HLB) medium was used to culture *N.daqingense* JX313^T^ with an optimal NaCl concentration (17.5%, *w*/*v*) [34]. When an additional 5% salt stress was applied, the NaCl concentration was adjusted to 22.5% (*w*/*v*) and the medium was named HLB+. The culture conditions were maintained at both 35 °C and pH 10.0. The culture conditions for *N. daqingense* LND5 differed from the aforementioned ones in that the NaCl concentration was 3% (*w*/*v*) and the pH was adjusted to 9.0. *E. coli* DH5α was cultured in LB medium (10 g/L of tryptone, 5 g/L of yeast extract, and 10 g/L of NaCl) at 37 °C and pH 7.0. The antibiotic concentrations used for selection in this study were ampicillin 100 μg∙mL^−1^, hygromycin B 50 μg∙mL^−1^, and erythromycin 200 μg∙mL^−1^.

### 4.2. Transcriptome Sequencing and Analysis

The transcriptome sequencing of *N. daqingense* JX313^T^ was based on two different NaCl concentrations. The NaCl concentration of the control group was 17.5% (*w*/*v*), while that of the salt-stressed group was 22.5% (*w*/*v*); each group had three replicates. *N. daqingense* was cultured under the above conditions until it reached the logarithmic growth phase, respectively. Then, 1 L of culture was collected for the extraction of total RNA. After removing tRNA, RNA was fragmented and a specific cDNA library was synthesized. The library fragments were enriched via PCR amplification and sequenced using the Illumina platform. The filtered data were aligned to the reference genome and the expression levels of genes were calculated. Based on this, the samples were further subjected to differential expression analysis, enrichment analysis, and cluster analysis.

### 4.3. Ultraviolet Mutagenesis of Low-Salt Adaptive Mutants

To obtain low-salt adaptive mutants, the bacterial solution was exposed, respectively, to UV light for 50 s, 60 s, 70 s, 80 s, 90 s, 100 s, 110 s, and 120 s under aseptic conditions. Each bacterial solution was then coated on an LB solid medium containing 10% NaCl (WT cannot grow in environments with a NaCl concentration less than 10%), with inoculated plates not exposed to UV light serving as the control group. Each experiment was performed in triplicate. After wrapping the plates with aluminum foil, they were incubated upside down at 35 °C for 7 days. Based on the number of single colonies on the plates, the lethality rates at different UV exposure times were calculated to determine the lethality curve. The exposure time with a lethality rate of about 70% was chosen for mutagenesis treatment. Mutants capable of maintaining their characteristics stably after 30 generations were screened. Their 16S rDNA was amplified using the 22F/1540R primers to eliminate bacterial interference. The optimal NaCl concentration and pH were determined for each mutant. The strain with the lowest optimal NaCl concentration was selected, and its growth curve was measured under optimal conditions.

### 4.4. Gene Knockout and Complementation

The gene knockout and complementation methods used in this study were improved according to the method of Wang et al. [48]; two common antibiotics were selected as selection markers. The gene knockout or complementation was achieved through spontaneous homologous recombination between the extremely halophilic archaea genome and prepared linear DNA fragments, traditionally known as the “gene replacement” method in archaeal gene manipulation [49]. The linear DNA fragment used for gene knockout consists of the following (from 5′ to 3′): the upstream homologous arm sequence (500 bp), erythromycin resistance sequence (735 bp), and downstream homologous arm sequence (500 bp). The linear DNA fragment used for gene complementation consisted of the following (from 5′ to 3′): the upstream homologous arm sequence (500 bp), hygromycin resistance sequence (1026 bp), target gene, and downstream homologous arm sequence (500 bp). The upstream and downstream homologous arms and target gene sequences were amplified via PCR using the gDNA as a template, and the antibiotic resistance sequences were amplified via PCR using the corresponding plasmids as a template. After purification, the PCR products and the pUC18 clone vector (linearized with *Bam*HI) were mixed gently (the addition amount of each component is 0.03 × length, ng; e.g., if the sequence length is 1000 bp, the required mass is 30 ng) and incubated at 50 °C for 30 min. After ligation, the samples were immediately cooled on ice for 3 min and then transformed into competent *E. coli* DH5α via the heat shock process. Through PCR identification, restriction enzyme digestion, and sequencing verification, the correct transformants with the correct connection sequence were selected. Recombinant plasmids were then extracted and used as templates for PCR amplification with the 5′ primer of the upstream homologous arm and the 3′ primer of the downstream homologous arm. Subsequently, the linear DNA fragments that can be used for gene knockout or complementation were acquired using gel extraction. The prepared linear DNA fragments were introduced into the recipient archaea via protoplast transformation. The obtained transformants were inoculated into 5 mL of HLB liquid medium and cultured at 35 °C and 180 rpm, for 7 days. The extracted gDNA of transformants were used as the template, and the pair of primers VY-FP/VY-RP was used to verify whether the knockout or complementation of the target gene was successful via PCR amplification. Then, the correct transformants were selected for sequencing verification.

### 4.5. Archaeal Protoplast Transformation

A total of 4.5 mL of archaeal broth (O.D._600nm_ 0.8) was enriched in a 2 mL tube via centrifuging at 5600× *g*. Then, the broth was gently resuspended in 1 mL of protoplast formation buffer (Solution I) and centrifuged at 5600× *g* for 2 min. The supernatant was removed, and the process was repeated twice. Then, the cells were gently suspended in 150 μL of protoplast formation solution (Solution II), combined with 15 μL of 0.5 M EDTA solution (pH 8.0), and left at room temperature (RT) for 10 min. An amount of 2 μg of the purified linear homologous fragment was added, gently mixed, and then left at RT for 15 min. A total of 175 μL of 60% PEG600 solution (preheated at 37 °C) was added, gently mixed, and then left at RT for 30 min. An amount of 1 mL of protoplast dilution (Solution III) was added to the tube along the wall to rinse the cells without mixing, then centrifuged at 3500× *g* for 2 min after standing at RT for 5 min; the supernatant was removed, and this process was repeated once. The cells were gently resuspended using 1 mL of protoplast regeneration solution (Solution IV), left at 37 °C for 2 h, and then revived at 37 °C and 40 rpm, for 8–12 h. An amount of 1 mL of protoplast transformation diluent (Solution V) was added along the tube wall to rinse the cells, and then mixed gently and centrifuged at 3500× *g* for 2 min. The supernatant was removed and this process was repeated once. Finally, the cells were resuspended with 200 μL Solution V, coated on HLB solid medium containing the corresponding antibiotics, and cultured at 37 °C for 7–10 days. The solution formulation used for protoplast transformation is shown in Table 3.

### 4.6. Determination of Intracellular Vitamin B_12_

The method of obtaining intracellular substances in this study was modified on the basis of “bacterial milking” by Nagata et al. [50]. A total of 1 L of fermentation broth was enriched in a 50 mL tube, centrifuged at 13,400× *g*, at 4 °C, for 10 min, and then the supernatant was discarded. The cells were rinsed with 50 mL of PBS buffer (precooled at 4 °C) and centrifuged at 13,400× *g*, at 4 °C, for 10 min. The supernatant was discarded and this process was repeated 3 times before being weighed. Under a strict dark environment, 1 g of wet-weight cells were quickly weighed in a new precooled 50 mL tube, resuspended in 20 mL ddH_2_O (precooled at 4 °C), vortexed for 15 s, and then stored at 4 °C for 1 h. After that, the cells were centrifuged at 13,400× *g* for 30 min at 4 °C under a dark environment. The supernatant was aspirated to a new precooled tube for HPLC detection.

A Waters Symmetry C18, 4.6 mm × 250 mm, 5 µm water-resistant column was used as the chromatographic column. Methanol/0.028 M Na_2_HPO_4_ solution (26:74, pH adjusted to 3.5 with phosphoric acid) was used as the mobile phase. The column flow rate was 1.0 mL/min, and the column temperature was 30 °C. The detection wavelength was 361 nm, and the injection volume was 10 µL. The stopping time was 10 min. The concentration of vitamin B_12_ in the sample was calculated based on the peak area, and the content of vitamin B_12_ per liter of fermentation broth was subsequently calculated. Each sample was measured in triplicate, and the final results are presented as the mean ± SEM.

### 4.7. Standard Experimental Procedures and Bioinformatics Analysis

Genomic DNA extraction, plasmid extraction, homologous recombination of DNA, and purification and recovery of DNA were carried out following the methods described by Wang et al. [38]. The determination of cell survival was carried out using Cell Counting Kit-8 (APExBIO K1018). The biomass is expressed as the O.D._600nm_ value measured using a UV spectrophotometer or the O.D._650nm_ value measured using a microplate reader. The primers utilized in this study are detailed in Table 4. DNA sequencing services were provided by RuiBiotech Institute (Beijing, China). RNA was extracted and reverse-transcribed using Bacteria RNA Extraction Kit (Vazyme R403) and HiScript II Q RT SuperMix for qPCR Kit (Vazyme R222), respectively. The RT-qPCR reaction system is shown in Table 5, and the reactions were performed in the following order: predenaturation at 95 °C for 5 min, then 95 °C for 10 s, followed by 60 °C for 30 s (40 cycles), and then the melting curve was obtained. Data were analyzed by using the 2^−ΔΔCT^ method to quantify gene expression levels, and the fold change in differentially expressed genes (DEGs) after salt stress was calculated.

The optimal NaCl concentration, optimal pH, cell survival, biomass, growth curve, and RT-qPCR experiments were all conducted with three replications, with each replication containing three samples. For comparisons between the two groups, an unpaired T-test was used for data analysis. For comparisons among three or more groups, ANOVA along with Tukey’s multiple comparison test was applied. The alignment of DNA sequence was conducted via the BLAST resource provided by the National Center for Biotechnology Information (NCBI) website https://blast.ncbi.nlm.nih.gov/Blast.cgi (accessed on 1 October 2023) The Volcano plot, M versus A (MA) plot, and gene clustering analysis plot were plotted using the ggplots2 software package of Rstudio 2022, and biclustering analysis was performed using the Pheatmap software (1.0.12) package of Rstudio 2022. Functional enrichment analysis of differentially expressed genes was conducted using the GO database http://geneontology.org/ (accessed on 1 July 2022) and the KEGG database https://www.kegg.jp/ (accessed on 1 July 2022).

## 5. Conclusions

In this study, transcriptome sequencing revealed the important role of vitamin B_12_ in the response of *N. daqingense* to salt stress. The addition of 10 mg/L of exogenous vitamin B_12_ significantly enhanced cell survival and biomass in *N. daqingense* under both optimal and salt-stressed conditions. The experimental validation showed that *N. daqingense* can resist salt stress by either the uptake of vitamin B_12_ from the environment or synthesizing it internally, which suggests that vitamin B_12_ acts as a specific compatible solute effector in the response of *N. daqingense* to salt stress.

## Figures and Tables

**Table 1:** Differentially expressed genes (partial).

Gene ID	Name	Fold Change	log_2_ (Fold Change)	*p*-Value
BB347_RS03400	*cbiE*	2.7626	1.4660	0.0049
BB347_RS03395	*cobH*	2.3105	1.2082	0.0392
BB347_RS03460	*cobC*	2.0859	1.0607	0.0141

**Table 2:** Strains and plasmids employed in the current study.

Strain or Plasmid	Description	Source or Reference
***S*** **trains**		
*N. daqingense* JX313^T^	Wild-type strain, an extremely halophilic archaea	Isolated and identified by our lab [31]
*N. daqingense* LND5	Low-salt adaptive mutant, obtained through UV mutagensis	This study
*N. daqingense* Δ*cobC*	The *cobC* gene-knockout strain, vitamin B_12_ biosynthesis deficiency	This study
*N. daqingense* +*cobC*	The *cobC* gene-complementation strain	This study
*E. coli* DH5α	Host strain for cloning	Vazyme Biotech Co., Ltd., Nanjing, China
**Plasmids**		
pUC18	Cloning vector, Amp^R^	Comate Biosciences Co., Ltd., Changchun, China
E-pUC57	Conferring erythromycin resistance, Amp^R^ and Erm^R^	Comate Biosciences Co., Ltd., Changchun, China
pSilentI	Conferring hygromycin resistance, Amp^R^ and Hyg^R^	MiaoLing Plasmid Platform, Wuhan, China
pUC-*cobC*-ko	Preparation of linear DNA fragments for cobC gene knockout, Amp^R^ and Erm^R^	This study
pUC-*cobC*-c	Preparation of linear DNA fragments for cobC gene complementation, Amp^R^ and Hyg^R^	This study

**Table 3:** Solution formulation used for protoplast transformation.

Component	Solution I	Solution II	Solution III	Solution IV	Solution V
NaCl	1 M	1 M	2.5 M	2.5 M	2.5 M
KCl	27 mM	27 mM	56 mM	56 mM	56 mM
Sucrose	37.5 g	150 g	150 g	150 g	37.5 g
Tris-HCl	50 mM (pH 8.5)	50 mM (pH 7.5)	50 mM (pH 7.5)	50 mM (pH 7.5)	50 mM (pH 7.5)
MgSO_4_	-	-	150 mM	134 mM	134 mM
CaCl_2_	-	-	3.75 mM	3 mM	3 mM
MgCl_2_	-	-	-	150 mM	150 mM
Total	1 L	1 L	1 L	1 L	1 L

**Table 4:** Primers used in this study.

Primers	Description	Amplicon Size (bp)	Sequence (from 5′ to 3′)
22F	Archaea 16S rDNA [46]	1500	ATTCCGGTTGATCCTGC
1540R			AGGAGGTGATCCAGCCGCAG
M13-47F	Sequencing primers of pUC18	Depends on the size of inserted fragment	CGCCAGGGTTTTCCCAGTCACGAC
M13R			CACACAGGAAACAGCTATGAC
VF-FP	To clone *cobC* upstream homologous arm sequence for gene knockout	500	TATGACCATGATTACGAATTCACACGCCGGTCGGCATCG
VF-RP			TTCTCGTTCATTTGCCCACCTCTTCGACGA
VE-FP	To clone erythromycin resistance sequence for gene knockout	735	GGTGGGCAAATGAACGAGAAAAATATAAAACACAGTCA
VE-RP			GTGGTAGTGTATCGAAACCGTTACTTATTAAATAATTTATAGCTATTGAAAAGAGA
VR-FP	To clone *cobC* downstream homologous arm sequence for gene knockout	500	CGGTTTCGATACACTACCACGA
VR-RP			CAGGTCGACTCTAGAGGATCCGCGTCCGGATCGCTCGAG
VY-FP	To confirm the success of gene knockout or complementation	2225 (knockout) 3233 (complementation)	GTCGACTTCGACGTGATCC
VY-RP			AGCGCGTCGACGTAG
VBF-RP	To clone *cobC* upstream homologous arm sequence for gene complementation, with VF-RP	500	GGCTTTTTCATTTGCCCACCTCTTCGACGA
VH-FP	To clone hygromycin resistance sequence for gene complementation	1026	GGTGGGCAAATGAAAAAGCCTGAACTCACCG
VH-RP			GTGTCTCGCTCATCTATTCCTTTGCCCTCGGACG
VBR-FP	To clone *cobC* and downstream homologous arm sequence for gene complementation, with VR-FP	1743	GGAATAGATGAGCGAGACACAGCCCAC
*cbiE*-FP	To detect differentially expressed genes	135	TGCTGACCTGTGGCTACAAG
*cbiE*-RP			GTACCTTCCCGACGAACTGG
*cobH*-FP		109	TGAGACGAGCATGGACATCG
*cobH*-RP			CAGGTGCTGGAACTCGATGT
*cobC*-FP		139	TTCGGGAGTTGATCGACGAC
*cobC*-RP			TCCCCGCAGTTCTCGTAGA
16S-FP	As reference gene for RT-qPCR	162	GCCGATTAGGTAGACGGTGG
16S-RP			GAGTCCCCTTATCGCACTCG

**Table 5:** RT-qPCR reaction system.

Component	Volume
Primer 1 (10 μM)	0.4 μL
Primer 2 (10 μM)	0.4 μL
2× AceQ qPCR SYBR Green Master Mix	10 μL
50× ROX Reference Dye 1	0.4 μL
cDNA	1 μL
ddH_2_O	up to 20 μL
Total	20 μL

## Data Availability

RNA-seq sequencing data have been deposited in SRA under accession number PRJNA1097806.
